# Viability and Motility of *Escherichia coli* Under Elevated Martian Salt Stresses

**DOI:** 10.3390/life14121526

**Published:** 2024-11-21

**Authors:** Max Riekeles, Berke Santos, Sherif Al-Morssy Youssef, Dirk Schulze-Makuch

**Affiliations:** 1 Astrobiology Group, Center of Astronomy and Astrophysics, Technical University Berlin, 10623 Berlin, Germany; berkesantos@gmail.com (B.S.); s.youssef@campus.tu-berlin.de (S.A.-M.Y.); schulze-makuch@tu-berlin.de (D.S.-M.); 2 Instituto Superior Técnico, Universidade de Lisboa, 1049-001 Lisboa, Portugal; 3 Section Geomicrobiology, German Research Centre for Geosciences (GFZ), 14473 Potsdam, Germany; 4 Department of Plankton and Microbial Ecology, Leibniz Institute of Freshwater Ecology and Inland Fisheries, 16775 Stechlin, Germany

**Keywords:** martian salts, perchlorate, chlorate, chloride, viability, microbial motility, life detection, prokaryotes, microscopy, biosignature

## Abstract

This study investigates the effects of three Martian-relevant salts—sodium chlorate, sodium perchlorate, and sodium chloride—on the viability and motility of *Escherichia coli*, a model organism for understanding microbial responses to environmental stress. These salts are abundant on Mars and play a crucial role in forming brines, one of the few sources of stable liquid water on the planet. We analyze the survivability under different salt concentrations using colony plating. Additionally, we perform a semi-automated motility analysis, analyzing microbial speeds and motility patterns. Our results show that sodium perchlorate is the most toxic, followed by sodium chlorate, with sodium chloride being the least harmful. Both survivability and motility are affected by salt concentration and exposure time. Notably, we observe a short-lived increase in motility at certain concentrations, particularly under sodium chlorate and sodium perchlorate stress, despite rapid declines in cell viability, suggesting a stress response mechanism. Given that motility might enhance an organism’s ability to navigate harsh and variable environments, it holds promise as a key biosignature in the search for life on Mars.

## 1. Introduction

When evaluating the habitability of present-day Mars, it is essential to consider several environmental factors, with the availability of water being particularly significant [1]. Although geomorphological evidence indicates that Mars once had large bodies of liquid water, the planet’s current conditions make liquid water largely unstable on its surface [2,3]. There are two main reasons why salts might be vital for the potential existence of life on Mars: firstly, they cause extreme freezing-point depressions [4,5]. Secondly, they allow for deliquescence—a process in which hygroscopic salts absorb moisture from the atmosphere and dissolve in it, leading to the formation of brines, as it has been investigated through various simulations in the laboratory and through theoretical models [6,7,8,9,10,11,12,13,14,15,16,17]. This means that liquid water could still exist, albeit temporarily, in the form of liquid brines, either on the Martian surface or near it. In this work, we investigate the immediate salt stress responses of *Escherichia coli* (*E. coli*) bacteria under different concentrations of sodium chloride (NaCl), sodium chlorate (NaClO_3_), and sodium perchlorate (NaClO_4_), all of which might play a crucial role in enabling putative life on Mars. Hygroscopic salts, including perchlorate (ClO4−) salts, have been detected on Mars in concentrations of 0.4–0.6 wt% at the Phoenix landing site [18,19]. Beyond perchlorate, chlorate—a stable intermediate oxychlorine species—also appears to be present on Mars. This is suggested by data from the Sample Analysis at Mars (SAM) instrument on the Curiosity rover at Gale Crater [20,21] and by the discovery of chlorate in the Martian meteorite EETA79001 [22]. Deposits of chloride, chlorate, and perchlorate salts or other hygroscopic substances could serve as a crucial source of liquid water in the Martian near-surface environment [23]. Sodium chloride, Earth’s most abundant salt, has also been detected in Martian meteorites [24], and widespread chloride deposits across the Martian surface have been observed [25,26]. Additionally, spectral analyses have revealed irradiated halite on Mars, further confirming the presence of salts on the planet [27]. There has been research regarding the growth of procaryotes in the salts that occur on Mars, and this work aims to extend this by including the effects on microbial motility [17,28].

If life is present on Mars, it would have needed to adapt to the planet’s shifting environmental conditions and increasing dryness, eventually depending on liquid brines as one of the few remaining viable habitats [29]. We propose that microbial motility could have been a critical factor in this adaptation, as it has independently evolved at least 18 times on Earth [30].

Motility, as an evolutionary trait, seems to be critical for microbes to take up nutrients, especially in a nutrient-poor environment. Although Brownian motion is gravity-independent, experiments in microgravity have shown that fluids surrounding bacterial cells remain mostly stagnant, therefore reducing mass transfer. Bacteria that are able to move are presumed to stir up the quiescent boundary layer around the cell and promote nutrient uptake [31,32,33]. Gravitational acceleration on Mars’ surface is only 3.72 m/s^2^ (38% of Earth’s gravity) [34]. Therefore, natural mixing processes like convection and sedimentation are reduced on Mars, making microbial motility a particularly useful characteristic in this type of environment. Motility, in general, offers significant advantages, such as escaping harmful environments and actively seeking out more favorable conditions, including accessing viable brines.

The motility of *E. coli* has been extensively studied [35,36,37,38,39,40,41,42], including under simulated microgravity [43]. It is characterized by a run-and-tumble motion, in which the microbes move toward a certain direction (with a mean runtime of about 1 s), and by a short stationary phase, in which it changes direction for the next run phase [44]. Different prokaryotic motility patterns have been described both qualitatively and quantitatively across various species and depend on environmental factors such as availability of nutrients and temperature [45,46,47]. Chemotaxis is the stimulation and alteration of motility due to chemical influences. *E. coli*, like many other species, has a biphasic response to signal molecules and whether a chemical substance acts as an attractor or repellent can depend on the concentration gradient [48,49]. The influence of chloride salts on microbial motility, including *E. coli*, has been researched [50,51]. Notably, over a century ago, Pfeffer discovered that some bacteria are attracted to inorganic salts [52]. This research extends previous studies by simultaneously examining viability and motility and by analyzing their interrelation. It broadens the scope beyond sodium chloride to include the Mars-relevant salts sodium perchlorate and sodium chlorate. 

Every planetary mission so far has used optical equipment for direct imaging, but it has mainly been used for geological purposes and not for the direct observation of putative microorganisms [53]. A significant challenge in using microscopic in situ observations to search for microbial organisms is the limited number of distinguishing features between cells and abiotic sediment particles. Clearly distinguishing primitive life forms like bacteria and archaea from mineral particles, even with the use of an electron microscope, is not always achievable [54]. Microbial motility is clearly distinguishable from random Brownian motion, making it an excellent biosignature [47,55,56,57,58,59]. Our research aims to investigate how Martian salts, with their ability to depress the freezing point and potentially allow for the existence of liquid water, interact with microbial motility.

## 2. Materials and Methods

### 2.1. Microorganisms Used

*E. coli* is the most extensively studied prokaryotic organism. This rod-shaped, gram-negative bacterium is a facultative anaerobe that inhabits the intestines of warm-blooded animals but can also thrive under a variety of other environmental conditions. The rods measure approximately 2 µm in length and 1 µm in diameter. *E. coli* is motile and propelled by flagella [60]. As a peritrichous bacterium, it possesses 5–10 flagella distributed randomly across its cell surface [61]. We used the *E. coli* K-12 wild-type (DSM 498) in our study which was obtained from the Leibniz Institute DSMZ—German Collection of Microorganisms and Cell Cultures GmbH (Braunschweig, Germany).

### 2.2. Incubation of Microorganisms

Before conducting microscopic observations and plating the microorganisms, the cells were prepared 48 h in advance in two stages. This method of incubation enabled the cells to grow into the stationary growth phase. Extractions from an initial cell stock culture were diluted (1:16) and incubated in standard nutrient broth 1 medium (Carl Roth, Karlsruhe, Germany) for 24 h at 25 °C. This was followed by a further dilution (1:5) and incubation in M9 minimal salts medium (Thermo Fischer Scientific, Waltham, MA, USA) for another 24 h at 25 °C. Following incubation, the cells were distributed in a dilution series according to the relative concentrations of salts. We tested sodium chloride, sodium chlorate, and sodium perchlorate in four regular intervals throughout set times of 0.01 h, one hour, four hours, and 24 h. Selecting four concentrations enabled the gradual observation of the impact of salt-rich fluids on microbes. The upper limits were selected in accordance with the last visible signs of motility during microscopic observations at any time points, leading to salt concentrations of 0.2 M, 0.4 M, 0.6 M, and 0.75 M for sodium chloride and of 0.25 M, 0.5 M, 0.75 M, and 1 M for both sodium perchlorate and sodium chlorate.

### 2.3. Plating

To assess cell survivability (i.e., overall viability), we plated each sample after their incubation with the various tested salts. Agar-based plates were utilized in sets of biological triplicates. The microbes were then extracted from the different salt concentrations at different timepoints and diluted in M9 minimal salts medium (Thermo Fischer Scientific, Waltham, MA, USA) with 50 µL plated on agar plates with standard nutrient broth 1 medium (Carl Roth, Karlsruhe, Germany). The plates were then incubated at 35 °C for 24–48 h. Following their incubation, we counted the number of colonies and calculated the concentration of Colony-Forming Units (CFUs) per mL. We calculated the fraction of surviving cells by dividing the average CFUs/mL of each sample by the average of the respective control (i.e., the controls were also plated at each one of the four timepoints tested). This fraction of survivability relative to control systematically accounted for changes in viability that were not correlated to the presence of any of the three salts tested in the samples. This normalization accounts for changes in survivability not caused by the salts.

### 2.4. Tracking and Analysis of Microbial Motility

For the recordings, 10 µL of sample was placed on a Neubauer Counting Chamber with a depth of 10 µm (Carl Roth, Karlsruhe, Germany). The observations were made using a Primo Star Full Köhler phase contrast microscope equipped with a 40× objective (ZEISS, Oberkochen, Germany). Attached to the microscope was a ZEISS Axiocam 105 color camera (ZEISS, Oberkochen, Germany) that streamed images directly to a computer. We utilized biological triplicates and each of these triplicates was recorded three times (technical triplicates) using the ZEISS ZEN 2 lite software (ZEISS, Oberkochen, Germany). Each recording lasted at least ten seconds, with frame rates of approximately 7 Hz (the exact frame rate of each recording was used in the further software analysis), and was saved as set of tif images.

**Figure 1:**
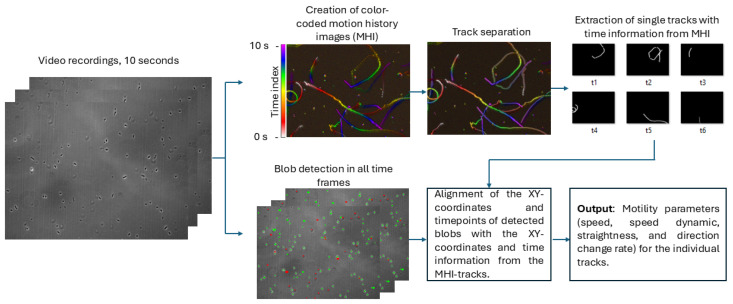
Workflow of the tracking methodology. The microscopic images have a resolution of 2560 × 1920 pixels, covering a field of view of 141 µm × 106 µm. This workflow allows for the accurate tracking of microbial paths that are overlapping, even in datasets with a low signal-to-noise ratio. This approach is based on the work of [59,62]. For further details, see Supplementary Materials.

We processed the recorded videos using the HELM software, which creates motion history images of the moving particles [62]. The software (version 3.1) is available at https://github.com/JPLMLIA/OWLS-Autonomy (accessed on 23 November 2023). Together with blob detection in the single timeframes, we were able to track the microbes with high accuracy. The workflow is shown in Figure 1, and the methodology is described in detail in [59]. For further details, see Supplementary Materials.

The relative fraction of motile cells was obtained by first calculating the fraction of motile cells in each sample. We obtained this fraction by counting the number of tracks observed in the motion history images and by dividing this value by the number of blobs detected on the first frame of each recording. We then calculated the fraction of motile cells for all the salt samples, as well as for the respective controls. The control recordings were obtained from the same cell culture on the same day as the sample observation, allowing us to account for differences in the fraction of motility due to slight environmental changes like temperature or a decline in nutrient availability (most notable after 24 h). The relative fraction of motile cells of a given sample is the ratio of the fraction of motile cells of that sample to the fraction of its corresponding control sample.

We analyzed the microbial movement in the samples containing the highest salt concentrations where motility was still observed after 1 h: 0.6 M sodium chloride, 0.75 M sodium chlorate, and 0.5 M sodium perchlorate. These results were compared to the control samples without salts, recorded within the hour preceding the salt tests. Additionally, we analyzed the effect of varying salt concentrations on individual motility at the 1 h timepoint for each of the three different salts. Moreover, we examined each salt’s maximum motility-sustaining concentration (including at the 24 h timepoint), focusing on differences in motion speed and patterns. This concentration was 0.4 M NaCl. We assessed microbial speed by calculating the mean velocity and the speed variation of individual microbes (referred to as “speed dynamic”). Additionally, we measured the direction change rate, defined as the number of significant directional shifts (>30°) per second. Lastly, we calculated the straightness index, which is the ratio of the net displacement to the total path length and is used as an indicator of movement efficiency.

## 3. Results

### 3.1. Effects of Sodium Chloride on Viability

**Figure 2:**
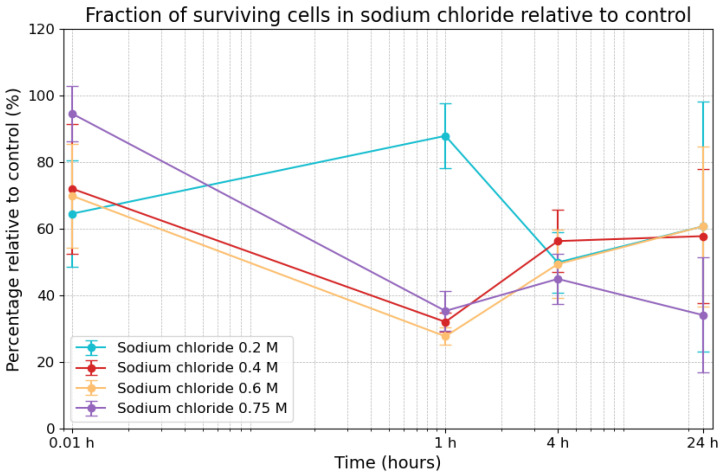
Fraction of surviving cells in sodium chloride compared to control. The error bars indicate the combined standard error of the mean. Time is plotted on a logarithmic scale on the *x*-axis.

The decline of cell survivability due to sodium chloride is shown in Figure 2. Under 0.2 M sodium chloride, the initial survivability was 64.5% (±15.9%) at the 0.01 h timepoint, increasing to 87.9% (±9.7) after 1 h, before declining to 49.9% (±9.2%) after 4 h and slightly increasing to 60.7% (±37.5%) by the 24 h timepoint. Under 0.4 M, survivability commenced at 72.0% (±19.5%) at the 0.01 h timepoint, decreased to 32.0% (±2.8%) after 1 h, and then rose to 56.3% (±9.3%) after 4 h, remaining stable at 57.8% (±20.1%) after 24 h. Similarly, under 0.6 M sodium chloride, survivability began at 69.9% (±15.6%) at the 0.01 h timepoint, dropped to 27.7% (±2.6%) after 1 h, before increasing again to 49.4% (±10.2) after 4 h, finally reaching 60.7% (±24.1%) after 24 h. Under 0.75 M, survivability was at its highest at the 0.01 h timepoint (94.6% ± 8.4%), decreasing to 35.3% (±6.0%) after 1 h and continuing to decline to 44.9% (±7.4%) after 4 h, reaching 34.1% (±17.2%) at the 24 h timepoint. The fraction of surviving cells in sodium chloride showed a consistent trend across all concentrations, with three notable exceptions: at the immediate timepoint (i.e., 0.01 h), the highest concentration (0.75 M) exhibited the largest fraction of surviving cells, while, at the 24 h timepoint, it exhibited the smallest. Additionally, at the 1 h timepoint, the lowest concentration (0.2 M) exhibited a significantly higher fraction of surviving cells compared to the other concentrations at the same timepoint.

### 3.2. Effects of Sodium Chlorate on Viability

**Figure 3:**
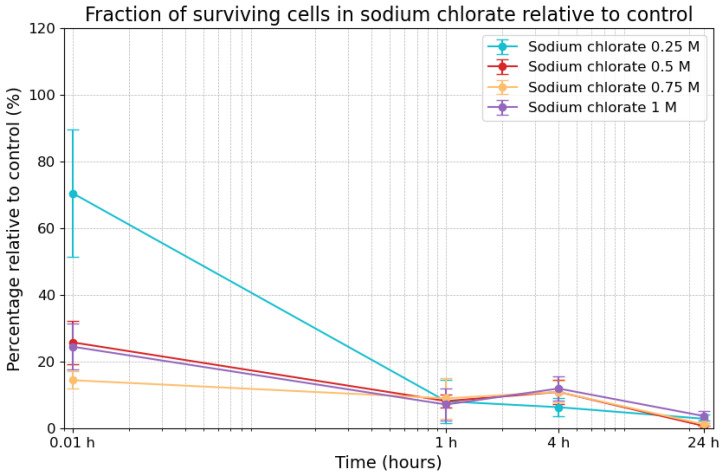
Fraction of surviving cells in sodium chlorate compared to control. The error bars indicate the combined standard error of the mean. Time is plotted on a logarithmic scale on the *x*-axis.

The survivability of cells exposed to varying concentrations of sodium chlorate over 24 h was measured as a percentage relative to the control. The results are shown in Figure 3. Under 0.25 M sodium chlorate, cell survivability was 70.5% (±19.1%) immediately after the start of the experiment (0.01 h), but it decreased rapidly to 8.1% (±6.5%) after 1 h. Survivability continued to decline, reaching 6.3% (±2.8%) after 4 h and 2.9% (±1.3%) after 24 h. In the 0.5 M condition, survivability was lower, starting at 25.8% (±6.5%) at the 0.01 h timepoint and remaining relatively constant at around 8.2% (±2.1%) after 1 h and even slightly increasing to 10.9% (±3.6%) after 4 h, before dropping to 0.72% (±0.0%) after 24 h. Under 0.75 M sodium chlorate, initial survivability was 14.4% (±2.6%) at the 0.01 h timepoint, followed by a decrease to 9.0% (±6.1) after 1 h, a slight increase to 10.9% (±3.3%) after 4 h, and a drop to 1.2% (±0.3%) a 24 h. Under the highest concentration (1 M), survivability began at 24.5% (±6.8%) at the 0.01 h timepoint, decreased to 7.2% (±4.8%) after 1 h and even increased slightly to 11.9% (±3.7%) after 4 h, before declining to 3.7% (±1.5%) after 24 h. Overall, the values across the different concentrations were quite consistent, except for a single outlier: the survivability estimate at the 0.01 h timepoint observed under the lowest tested concentration (0.25 M), i.e., 70.5%

### 3.3. Effects of Sodium Perchlorate on Viability

**Figure 4:**
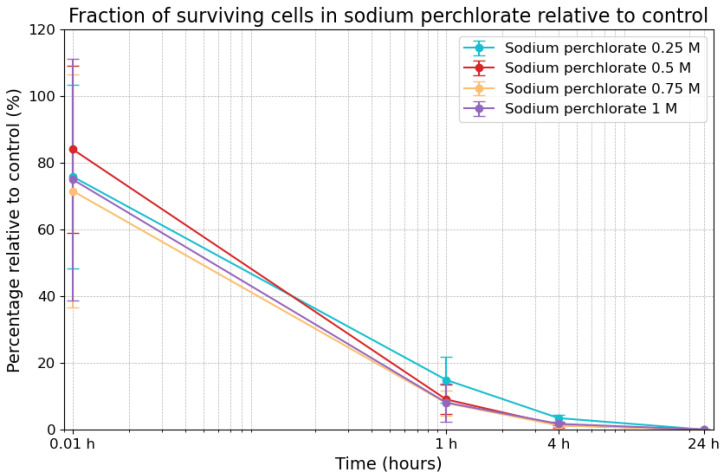
Fraction of surviving cells in sodium perchlorate compared to control. The error bars indicate the combined standard error of the mean. Time is plotted on a logarithmic scale on the *x*-axis.

Exposure to sodium perchlorate also caused a decline in cell survivability over time, as shown in Figure 4. Under 0.25 M sodium perchlorate, survivability was initially 75.9% (±27.6%) at the 0.01 h timepoint. It decreased to 14.9% (±6.8%) after 1 h and to 3.4% (±1.1%) after 4 h, with complete cell death (0%) occurring at the 24 h timepoint. Under 0.5 M, survivability was slightly higher at the 0.01 h timepoint (84.1% ± 25.2%) but dropped to 9.0% (±4.5%) after 1 h and to 1.3% (±0.8%) after 4 h, reaching 0% after 24 h. Similarly, under 0.75 M, survivability started at 71.6% (±35.0%) at the 0.01 h timepoint, dropped to 8.0% (±3.8%) after 1 h, and fell to 1.2% (±0.4%) after 4 h, before zeroing out at the 24 h timepoint. Under the highest concentration (1 M), survivability began at 75.0% (±36.2%) at the 0.01 h timepoint, then decreased to 8.1% (±5.6%) after 1 h and to 1.7% (±0.5%) after 4 h, before reaching 0.01% after 24 h. Overall, the fraction of surviving cells in sodium perchlorate followed a similar trend across all concentrations.

### 3.4. Effects of Sodium Chloride on Motility

**Figure 5:**
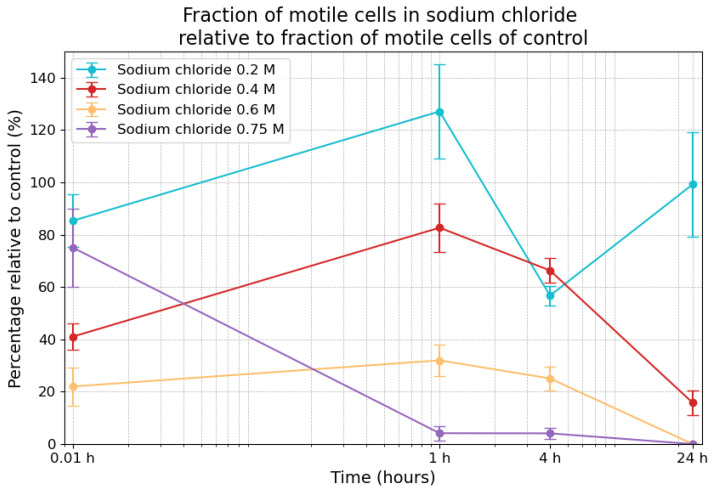
Fraction of motile cells in sodium chloride relative to the fraction of motile cells in the control measurements. The error bars indicate the combined standard error of the mean. Time is plotted on a logarithmic scale on the *x*-axis.

*E. coli* displayed the following motility alterations following exposure to sodium chloride, as shown in Figure 5. At the 0.01 h timepoint, motility in the 0.2 M sodium chloride condition was 85.3% (±10.0%) compared to the control, and it was followed by an increase to 127.1% (±18.1) after 1 h. By the 4 h timepoint, motility had decreased to 56.7% (±3.7%), but it increased to 99.2% (±20.0%) after 24 h. Under 0.4 M sodium chloride, motility was 41.1% (±4.9%) at the 0.01 h timepoint, increasing to 82.7% (±9.3%) after 1 h, before decreasing to 66.3% (±4.7%) after 4 h and to 15.8% (±4.8%) after 24 h. In the 0.6 M sample, motility was recorded to be 22.0% (±7.3%) at the 0.01 h timepoint, with increased motility at the 1 h timepoint (32.0% ± 6.1%), dropping to 25.1% (±4.6%) by the 4 h mark and to 0% by the 24 h timepoint. The highest concentration of sodium chloride (0.75 M) led to a 75.1% (±15.0%) fraction of motility at the 0.01 h timepoint, followed by a relative fraction of 4.1% (±2.7%) motility at the 1 h timepoint and a 4.1% (±2.1%) fraction at the 4 h timepoint, with no motility recorded after 24 h. The combination of the survivability data and the fraction of motile cells allowed for the calculation of the fraction of motile cells among the surviving cells in the sodium chloride samples, shown in Table 1. The fraction of motile cells in the control was between 4% and 8%.

### 3.5. Effects of Sodium Chlorate on Motility

**Figure 6:**
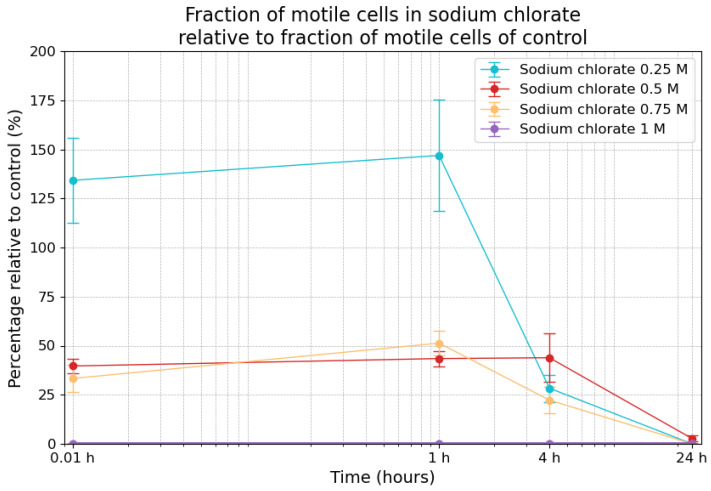
Fraction of motile cells in sodium chlorate relative to the fraction of motile cells in the control measurements. The error bars indicate the combined standard error of the mean. Time is plotted on a logarithmic scale on the *x*-axis.

The motility of the cells exposed to different concentrations of sodium chlorate over 24 h was measured relative to the fraction of motile cells in the control samples (Figure 6).

At the 0.01 h timepoint, the motility of the cells in the 0.25 M sodium chlorate sample was estimated at 134% (±21.6%), indicating a stimulation of motility by the salt. At the 1 h timepoint, motility increased to 146.9% (±36.4%), before decreasing to 28.3% (±6.9%) after 4 h and reaching 0% after 24 h. For the 0.5 M condition, the relative fraction of motility at the 0.01 h timepoint was 39.6% (±3.7%), which increased slightly to 43.4% (±3.9%) after 1 h, remaining relatively stable at 43.9% (±12.3%) after 4 h before dropping to 2.7% (±1.4%) after 24 h. When using a 0.75 M concentration, the fraction of motility was 33.3% (±6.9%) at the 0.01 h timepoint, followed by an increase to 51.3% (±6.2%) after 1 h and a drop to 22.3% (±6.8%) by the 4 h timepoint, eventually reaching 0% after 24 h. In contrast, with respect to the highest concentration of 1 M sodium chlorate, the fraction of motile cells was 0% at the 0.01 h, 4 h, and 24 h timepoints, and a statistically insignificant fraction of motile cells (one motile cell among approx. 5000 cells) was recorded at the 1 h timepoint.

The combination of the survivability results and the fraction of motile cells allowed for the calculation of motile cells among the surviving cells in the salt samples, shown in Table 2. While the fraction of motile cells in the control was 4.3% at the 1 h mark, the fraction of motile cells among survivors was considerably higher in the 1 h sample with respect to all concentrations except for the highest. This was evidenced by a *p*-value of 0.28 for 0.25 M sodium chlorate, 0.009 for 0.5 M sodium chlorate, and 0.22 for 0.75 M sodium chlorate, indicating a statistically significant difference relative to the 0.5 M concentration.

### 3.6. Effects of Sodium Perchlorate on Motility

**Figure 7:**
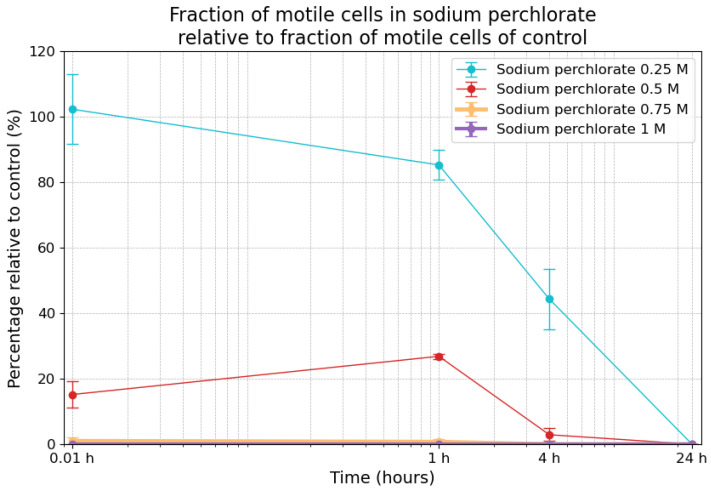
Fraction of motile cells in sodium perchlorate relative to the fraction of motile cells in the control measurements. The error bars indicate the combined standard error of the mean. Time is plotted on a logarithmic scale on the *x*-axis.

The variability of the relative fraction of motile cells caused by the exposure to sodium perchlorate is shown in Figure 7. At the 0.01 h timepoint, motility in the 0.25 M sodium perchlorate samples was 102.3% (±10.6%), indicating an initial increase compared to the control. However, motility progressively decreased to 85.3% (±4.5%) after 1 h, 44.3% (±9.2%) after 4 h, and, ultimately, reached 0% after 24 h. With respect to the 0.5 M condition, motility was 15.0% (±4.0%) at the 0.01 h timepoint, increasing to 26.8% (±0.8%) after 1 h but declining completely to 0% by the 4 h mark and remaining at 0% until the 24 h timepoint. For the 0.75 M sample, the fraction of motile cells relative to the control was only 1% after 1 h, 0.7% after 4 h, and 0% after 4 h and 24 h. The cells in the 1 M sodium perchlorate samples exhibited only statistically insignificant motility at the 1 h timepoint (one motile cell detected among approximately 5000 cells), while, with respect to the remaining timepoints, no motility was detected at all. The fraction of motile cells among the surviving cells in the sodium perchlorate samples is shown in Table 3. While the fraction of motile cells in the control was between 7% and 11%, the fraction of motile cells among the survivors was considerably higher at the 1 h and 4 h marks for 0.25 M and 0.5 M. The *p*-value for the 0.25 M concentration at the 4 h timepoint was 0.044 relative to the control, indicating statistical significance.

### 3.7. Motility Behavior of Individual Cells

**Figure 8:**
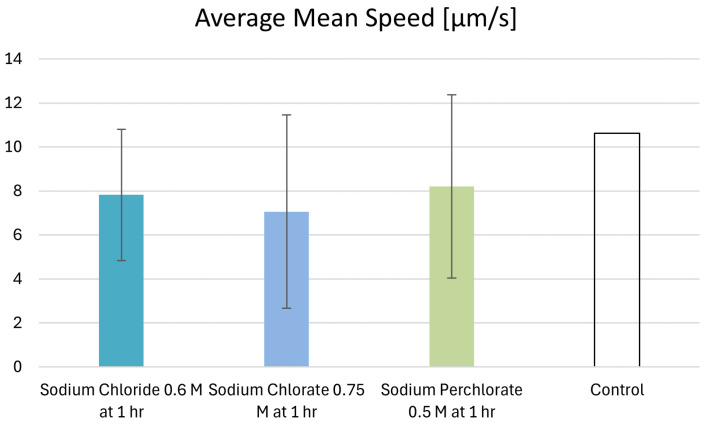
Average mean microbial speeds at the 1 h mark under maximum motility-sustaining salt concentrations. The control measurements showed, on average, higher mean speeds compared to the samples under the highest salt concentrations in which motility still occurred after 1 h. For each sample, an individual control analysis was performed, but only the total average of the controls was used for this figure. In relation to sodium chloride, we analyzed a total of 45 microbial tracks with a mean runtime of 7.1 s, and, in relation to its control, 69 microbial tracks exhibiting a mean runtime of 5.8 s were analyzed. In relation to sodium perchlorate, we analyzed 47 microbial tracks with a mean runtime of 8.0 s, and, for its respective control, 132 microbial tracks with a mean runtime of 7.9 s were analyzed. With respect to sodium chloride, we analyzed 39 tracks with a mean runtime of 7.6 s, and, in relation to its control, 63 microbes with a mean runtime of 7.8 s were analyzed. For further details, see Supplementary Material.

**Figure 9:**
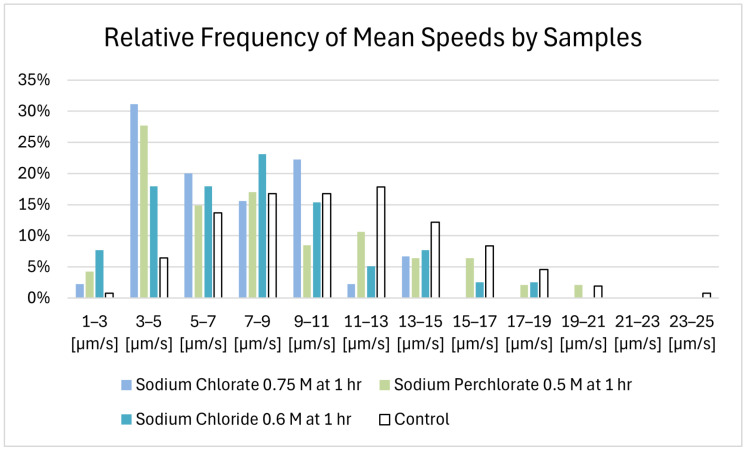
Histogram of the relative frequency of mean speeds by samples.

The individual cells of the control recording show, on average, a mean speed of 10.1 µm/s–11.2 µm/s, with standard deviations of 3.9–4.3 µm/s. The samples exhibiting salt-induced motility at the 1 h mark under maximum motility-sustaining salt concentrations exhibited a lower average mean speed (0.6 M sodium chloride: 7.8 µm/s; 0.75 M sodium chlorate: 7.1 µm/s; 0.5 M sodium perchlorate: 8.2 µm/s). However, the differences relative to the control were not statistically significant, though the comparison with respect to sodium chlorate approached significance. The comparison of the salt samples with the control counterparts led the 0.6 M sodium chloride samples to a t-value of 1.49 and a *p*-value of 0.16. With respect to the 0.6 M sodium chlorate samples, the values were t = 2.1 and *p* = 0.005. Lastly, with regard to sodium perchlorate, the values were t = 1.19 and *p* = 0.25. The average mean speeds for all samples are shown in Figure 8. The relative frequency distribution of the mean speeds is shown in Figure 9. In the control group, the largest percentage of microbes was observed moving at speeds between 11 µm/s and 13 µm/s (17.9%). A significant percentage was also observed between 7 µm/s and 9 µm/s (16.7%) and between 9 µm/s and11 µm/s (16.7%), showing that the majority of microbes in the control group fell within a moderate speed range. Some microbes (5.3%) in the control group reached a mean speed of 23–25 µm/s. The microbes in the sodium chloride solution showed a more uniform distribution, with no dominant peak. A small fraction in this sample was in the 17–19 µm/s range. The sodium chlorate sample showed a significant portion of microbes moving at relatively low speeds, with the highest percentage of 31% moving at 3–5 µm/s. Here, no microbe exhibited a mean speed higher than 15 µm/s. Similarly, the sodium perchlorate sample showed the largest fraction (28%) of microbes moving in the 3–5 µm/s range. No microbe moved faster than 19 µm/s.

The sample with the lowest average mean speed of the microbes (sodium chlorate 0.75 M) also exhibited the lowest speed dynamics, meaning that the individual microbes tended to accelerate and decelerate less compared to the microbes in the control samples. The sodium chloride and sodium perchlorate samples exhibited slightly higher speed dynamics, on average. Sodium perchlorate also exhibited the lowest direction change rate (and the highest straightness index), whereas the sodium perchlorate and sodium chloride samples exhibited a slightly higher direction change rate compared to the control. All the mentioned differences relative to the control were not significant. The t-values and *p*-values of the samples are provided in the Supplementary Material. All differences relative to the control were found to be not significant.

The comparison across the motility-sustaining salt concentrations at the 1 h timepoint (0.2 M, 0.4 M, and 0.6 M of sodium chloride, 0.25 M, 0.5 M, and 0.75 M of sodium chlorate, and 0.25 and 0.5 M of sodium perchlorate) showed, in relation to all three salts, that higher salt concentrations led to lower values of mean average speed. However, the differences were not statistically significant; neither were the results of the other motility parameters (details in the Supplementary Material).

The sample containing 0.4 M sodium chloride offered motility comparisons over all time points (0.01 h, 1 h, 4 h, and 24 h). Here, like in the previous samples, the mean average speeds showed no significant differences. Likewise, the other motility parameters showed no significant differences, although the lowered speed dynamics after 24 h approached statistical significance. The values of all motility parameters are shown in the Supplementary Material.

## 4. Discussion

The experiments show that sodium chloride, sodium chlorate, and sodium perchlorate all have deleterious effects on the survivability of *E. coli* cells, with sodium chloride being the least harmful and sodium chlorate and sodium perchlorate being notably more toxic. At a concentration of 0.75 M, sodium chloride allowed for higher survivability compared to both sodium chlorate and sodium perchlorate across all time points. Statistical analysis revealed a significant difference between sodium chloride and sodium chlorate at the 0.01 h timepoint (*p* = 0.002), while the difference between sodium chloride and sodium perchlorate was not statistically significant at this timepoint (*p* = 0.4). Further comparisons between sodium chloride and sodium chlorate showed significant differences at both the 1 h (*p* = 0.006) and the 4 h (*p* = 0.007) timepoints. Similarly, sodium chloride and sodium perchlorate exhibited statistically significant differences at the 1 h (*p* = 0.004) and 4 h marks (*p* = 0.009). By the 24 h timepoint, however, no statistically significant differences were observed between sodium chloride and either sodium chlorate or sodium perchlorate (both *p* = 0.08).

By comparing the 0.75 M sodium chlorate and the 0.75 M sodium perchlorate, it was possible to conclude that sodium chlorate was associated, generally, with higher survivability, with the exception of the 0.01 h timepoint where the difference was not statistically significant (*p* = 0.1). The comparison at the 1 h timepoint also showed no significant difference (*p* = 0.8). However, by the 4 h (*p* = 0.03) and 24 h (*p* = 0.02) marks, the differences between sodium chlorate and sodium perchlorate reached statistical significance. While the surviving fractions in sodium chlorate and sodium perchlorate were, under all concentrations, fewer than 5% after 24 h, the sodium chloride samples exhibited a higher survivability over the same period (with the lowest value of 34.1% (±17.2%) in the 0.75 M sample).

The rapid onset of cell death at higher concentrations of chlorate and perchlorate indicates their strong toxic properties. Exposure to sodium chlorate resulted in a rapid and substantial decline in cell viability across all tested concentrations. This pronounced toxicity is likely due to the oxidative properties of the chlorate ion which can disrupt essential cellular processes by inducing oxidative stress [63]. Sodium perchlorate exhibited comparable effects on *E. coli* viability, presumably also as a result of its oxidative properties [64]. Cell survivability decreased across all concentrations in the sodium chlorate and perchlorate samples, reaching a value of zero at the 24 h timepoint.

In contrast, sodium chloride had a less severe impact on cell viability. While there was an initial decrease in survivability at certain concentrations, the cells demonstrated a capacity for recovery over time.

Microbial motility was also altered by exposure to the tested salts: a short-term stimulation of motility was observed across all salt treatments, resulting in a higher fraction of motile cells compared to the control sample. Compared to the other salts, motility in the sodium chloride sample also fluctuated, but it did not decline to zero at the lower concentrations (0.2 M and 0.4 M) as observed from the 24 h recordings of all sodium chlorate and sodium perchlorate samples. An inverse relationship between viability and motility was found for sodium chloride, sodium chlorate, and sodium perchlorate in the first hour. The impact became even more striking when accounting for cell death (fraction of motile cells among the survivors). In the control samples, motile cells accounted for an average of 7% of the population, assuming that all cells had been alive at the start of the experiment. In contrast, the surviving cells in the salt treatments demonstrated notably higher motility rates, particularly in the short term and at lower salt concentrations. The fraction of motile cells among the survivors in sodium chloride was not significantly higher than that exhibited by the control samples, in contrast to the 0.5 M sodium chlorate sample at the 1 h mark and the 0.25 M sodium perchlorate sample at the 4 h mark. At the highest concentration (0.75 M), however, the sodium chloride sample exhibited a more drastic decrease in motility in the first hour compared to the other salt samples at this concentration in the same time span. However, due to the underlying uncertainty regarding the initial number of alive cells in the controls and the large standard deviations, these numbers could not be determined accurately. Nevertheless, for all three salts, a pattern was observed where motility increased from the immediate measurement after exposure (0.01 h) to the 1 h mark in several cases. This transient hypermotility can be explained by a stress response, where cells initially increase their movement to adapt or escape unfavorable conditions before succumbing to the toxic effects. The magnitude of the motility increased, and the viability drop varied depending on the salt and its concentration. The ability of *E. coli* to increase its motility in the short term as a response to stress from sodium chloride, sodium chlorate, and sodium perchlorate at different concentrations indicates that motility could aid the localization of microhabitats with more favorable conditions, such as subsurface niches with lower oxidative stress or higher water activity.

We observed that the maximum motility-sustaining salt concentrations for each salt at the one-hour mark were associated with lower average mean speeds. In particular, the slowest sample (0.75 sodium chlorate at the 1 h mark) also exhibited decreased speed dynamics and direction change rate, although no motility changes regarding speed or motility pattern were statistically significant. Further, very fast microbes (20–30 µm/s) did not appear in the salt samples, in contrast to the control samples. Additionally, with increasing salt concentrations, each salt generally caused a decrease in the average speed of the microbes. However, again, these changes, along with the variations in other motility parameters, were not statistically significant.

Although the motility behavior of individual cells in terms of speed and pattern did not show statistically significant alterations compared to the control samples, the 0.5 M sodium chlorate sample at the 1 h mark and the 0.25 M sodium perchlorate sample at the 4 h mark showed a significant increase in the fraction of motile cells among the surviving cells. One possible explanation for this could be the increased viscosity of the medium caused by the presence of salt. Salts in the solutions lead to a higher viscosity (1 M NaCl increases the viscosity of water by approx. 9% at 20 °C [65]) and it has also been shown before that elevated viscosity can increase the motility of various cell types, as a result of a biophysical mechanism [66]. Furthermore, osmotic stress induced by sodium chloride and the chaotropic and oxidative stress induced by both sodium perchlorate and sodium chlorate can be expected to have significant effects on motility behavior. It could also be partly explained by the fact that motile cells are particularly robust (they have enough energy not only to cover their basal maintenance costs, but also to be active). This may indicate that they have extra energy to perform life-sustaining activities such as repairing damaged proteins when exposed to salt stress. Noteworthy is the fact that salt stress can stimulate cells, especially suggested by the motile fraction in the 0.2 M sodium chloride sample at the 1 h mark and the motile fraction in the 0.25 M sodium chlorate sample at the 0.01 h and 1 h marks compared to their controls. This implies that not only do motile cells survive while some stagnant cells perish, but also that some stagnant cells transition to motility. It has been shown that, under poor carbon source conditions, a reduction in growth occurs and, at the same time, motility genes are expressed, providing a fitness benefit [67]. It is reasonable to think that a similar mechanism comes into play under salt stress.

## 5. Conclusions and Outlook

Our experiments using *E. coli* show the effects of increasing salt concentrations of sodium chloride, sodium chlorate, and sodium perchlorate on the viability of this bacterium. We show that sodium chloride is the most tolerated salt, followed by sodium chlorate and sodium perchlorate, both of which exert the most toxic effects. One additional parameter of viability is motility, which is rarely tested but is part of this study. While the exposure to the salts led, in some samples, to a significant increase in the fraction of motile cells compared to the controls, statistical significance could not be shown regarding the changes in speed and motility patterns of individual cells. More research is very much needed in this context, as motility enhances an organism’s ability to navigate harsh and variable environments. It holds promise as a key biosignature in the search for life on Mars, as substantial salt stresses is to be expected in Martian environments. While it may be challenging to encounter liquid water in the near-surface environment that is accessible to a landed spacecraft (i.e., the upper 2 m of Martian surface) [68], there are likely still near-surface environments, even in hyperarid regions, where liquid water exists. Potential habitats include (1) (meta)stable brines [16] (however, these brines would only exist at very low temperatures and for short durations when high humidity permits brine formation, presenting significant challenges for life), (2) deliquescent environments in NaCl-rich salt rocks, especially in Mars’ Southern Highlands [23], and (3) topographically low locations, such as at the bottom of Valles Marineris (or inside caves), where atmospheric pressures may be sufficient to support liquid (salty) water [69].

A deeper understanding of areas that may contain liquid water on Mars—especially those that are both accessible and feasible for landing—is essential to guide future space exploration missions. Equally important is a thorough evaluation of the instrumentation needed to detect and study such environments.

To achieve this, it is vital to assess the requirements for a sampling system capable of capturing brine samples and separating potential organisms from Martian sediments through physical or (bio)chemical methods. For optimal detection, the selection of an optical device is critical. One promising option is a digital holographic microscope [57,70]. A microscope design, which features minimal optical and mechanical components, would be suited to the analysis of sparse samples with resilience in extreme in situ conditions, as demonstrated on Earth [57,71]. Automated data analysis could then process in situ observations, transmitting only the most promising indicators of microbial motility back to Earth for further study [47,62].

Future developments of in situ life detection instruments should prioritize the detection of motility as a critical biosignature. Testing a broader range of organisms, especially extremophiles, could reveal whether motility is a generalizable trait that could increase the habitability potential of Mars.

## Figures and Tables

**Table 1:** Fraction of motile cells among the surviving cells in sodium chloride. This is based on the assumption that 100% of the control cells are alive. The standard deviations include the standard deviations of the fraction of motile cells in the samples, including the controls, as well as the standard deviations of the survivability results. The fraction of motile cells in the controls was between 4% and 8%.

Sodium Chloride Concentrations	0.01 h	1 h	4 h	24 h
0.2 M NaCl	8.4% (±2.4%)	9.2% (±1.8%)	9.0% (±1.8%)	5.9% (±3.9%)
0.4 M NaCl	3.2% (±1.0%)	14.5% (±2.3%)	9.6% (±1.8%)	1.5% (±0.7%)
0.6 M NaCl	2.0% (±0.8%)	7.5% (±1.7%)	4.2% (±1.2%)	0.0% (±0.0%)
0.75 M NaCl	5.0% (±1.2%)	0.7% (±0.5%)	0.7% (±0.4%)	0.0% (±0.0%)

**Table 2:** Fraction of motile cells among the surviving cells in sodium chlorate. This assumes that 100% of the control cells are alive. The standard deviations include the standard deviations of the fraction of motile cells in the samples, including the controls, as well as the standard deviations of the survivability results. The fraction of motile cells in the controls was between 4.4% and 11%.

Sodium Chlorate Concentrations	0.01 h	1 h	4 h	24 h
0.25 M NaClO_3_	9.2% (±3.0%)	87.6% (±72.5%)	26.6% (±13.8%)	0.0%
0.5 M NaClO_3_	17.6% (±4.9%)	60.9% (±16.6%)	24.6% (±10.9%)	43.5% (±22.3%)
0.75 M NaClO_3_	22.1% (±6.4%)	54.6% (±38.2%)	13.5% (±5.9%)	0.0%
1 M NaClO_3_	0.0%	0.0%	0.0%	0.0%

**Table 3:** Fraction of motile cells among the surviving cells in sodium perchlorate. This assumes that 100% of the control cells are alive. The standard deviations include the standard deviations of the fraction of motile cells in the samples, including the controls, as well as the standard deviations of the survivability results. The fraction of motile cells in the controls was between 7% and 11%. The calculated value of the 0.25 M concentration at the 4 h mark was 101% and was, here, corrected to 100%. For details, see Supplementary Material.

Sodium Perchlorate Concentrations	0.01 h	1 h	4 h	24 h
0.25 M NaClO_4_	14.5% (±5.6%)	61.6% (±28.7%)	100.0% (±38.7%)	0.0%
0.5 M NaClO_4_	2.0% (±0.8%)	33.3% (±16.7%)	17.3% (±15.6%)	0.0%
0.75 M NaClO_4_	0.2% (±0.2%)	1.0% (±1.1%)	0.0%	0.0%
1 M NaClO_4_	0.0%	0.0%	0.0%	0.0%

## Data Availability

The original data presented in the study are openly available in the DepositOnce repository at https://doi.org/10.14279/depositonce-21707.

## Supplementary Materials

The following supporting information can be downloaded at: https://doi.org/10.14279/depositonce-21707, Supporting Information for Viability and Motility of Escherichia coli under Elevated Martian Salt Stresses: Elaboration on (1) the motility parameters of all datasets, (2) the *p*- and t-values of the different data sets, (3) the tracking methodology, and (4) the statistical analysis. (Text S1, Figure S1, Tables TS1, TS2, and TS3); Dataset S0: Color palette used for tracking the MHI tracks; Dataset S1: Python file for creating single tracks from the drawn tracks in the MHI; Dataset S2: Python file for saving the manually tracked tracks as npy files; Dataset S3: Python file for fine-tuning the parameters of blob detection; Dataset S4: Python file for detecting the particles in each frame; Dataset S5: Python file for aligning MHI tracks and blob detection results; Dataset S6: Python file for copying all tracks into one place; Dataset S7: Python file for extracting acquisition frequencies; Dataset S8: Python file for converting XY-coordinates from pixels to µm, converting timeframes to seconds, and applying filtering of tracks; Dataset S9: Python file for motility analysis of all filtered tracks; Dataset S10: Python file for t- and *p*-value calculation; Dataset S11: CSV file containing data for Figure 2, Figure 3 and Figure 4; Dataset S12: Excel file with original CFU data; Dataset S13: Excel file with data for Figure 5, Figure 6 and Figure 7; Dataset S14: Excel file with data for Table 1, Table 2 and Table 3; Dataset S15: CSV file with motility data for 0.6 M sodium chloride at 1 h with control data; Dataset S16: CSV file with motility data for 0.75 M sodium chlorate at 1 h with control data; Dataset S17: CSV file with motility data for 0.5 M sodium perchlorate at 1 h with control data; Dataset S18: ZIP file containing automated tracking results of software for individual microbial tracks of sodium chloride, sodium chlorate, sodium perchlorate, and controls at 1 h; Dataset S19: ZIP file with MHIs and tracks of sodium chloride; Dataset S20: ZIP file with MHIs and tracks of sodium chlorate; Dataset S21: ZIP file with MHIs and tracks of sodium perchlorate.
